# Rhizofungus *Aspergillus terreus* Mitigates Heavy Metal Stress-Associated Damage in *Triticum aestivum* L.

**DOI:** 10.3390/plants13182643

**Published:** 2024-09-21

**Authors:** Naveen Dilawar, Muhammad Hamayun, Amjad Iqbal, Bokyung Lee, Sajid Ali, Ayaz Ahmad, Abdulwahed Fahad Alrefaei, Turki Kh. Faraj, Ho-Youn Kim, Anwar Hussain

**Affiliations:** 1 Department of Botany, Abdul Wali Khan University Mardan, Mardan 23200, Pakistan; naveen@awkum.edu.pk (N.D.); hamayun@awkum.edu.pk (M.H.); 2 Department of Food Science and Technology, Abdul Wali Khan University Mardan, Mardan 23200, Pakistan; amjadiqbal@awkum.edu.pk; 3 Department of Food Science and Nutrition, Dong-A University, Busan 602760, Republic of Korea; bolee@dau.ac.kr; 4 Department of Horticulture and Life Science, Yeungnam University, Gyeongsan 38541, Republic of Korea; 5 Department of Biotechnology, Abdul Wali Khan University Mardan, Mardan 23200, Pakistan; ahdayazb5@awkum.edu.pk; 6 Department of Zoology, College of Science, King Saud University, Riyadh 2455, Saudi Arabia; afrefaei@ksu.edu.sa; 7 Department of Soil Science, College of Food and Agriculture Sciences, King Saud University, Riyadh 2455, Saudi Arabia; talasiri@ksu.edu.sa; 8 Korea Institute of Science and Technology, Gangneung 25451, Republic of Korea; hykim@kist.re.kr

**Keywords:** abiotic stress, bio-stimulation, heavy metals, lead and copper stress, rhizofungus, stress mitigation

## Abstract

Industrial waste and sewage deposit heavy metals into the soil, where they can remain for long periods. Although there are several methods to manage heavy metals in agricultural soil, microorganisms present a promising and effective solution for their detoxification. We isolated a rhizofungus, *Aspergillus terreus* (GenBank Acc. No. KT310979.1), from *Parthenium hysterophorus* L., and investigated its growth-promoting and metal detoxification capabilities. The isolated fungus was evaluated for its ability to mitigate lead (25 and 75 ppm) and copper (100 and 200 ppm) toxicity in *Triticum aestivum* L. seedlings. The experiment utilized a completely randomized design with three replicates for each treatment. *A. terreus* successfully colonized the roots of wheat seedlings, even in the presence of heavy metals, and significantly enhanced plant growth. The isolate effectively alleviates lead and copper stress in wheat seedlings, as evidenced by increases in shoot length (142%), root length (98%), fresh weight (24%), dry weight (73%), protein content (31%), and sugar content (40%). It was observed that wheat seedlings possess a basic defense system against stress, but it was insufficient to support normal growth. Fungal inoculation strengthened the host’s defense system and reduced its exposure to toxic heavy metals. In treated seedlings, exposure to heavy metals significantly upregulated MT1 gene expression, which aided in metal detoxification, enhanced antioxidant defenses, and maintained metal homeostasis. A reduction in metal exposure was observed in several areas, including normalizing the activities of antioxidant enzymes that had been elevated by up to 67% following exposure to Pb (75 mg/kg) and Cu (200 mg/kg). Heavy metal exposure elevated antioxidant levels but also increased ROS levels by 86%. However, with *Aspergillus terreus* colonization, ROS levels stayed within normal ranges. This decrease in ROS was associated with reduced malondialdehyde (MDA) levels, enhanced membrane stability, and restored root architecture. In conclusion, rhizofungal colonization improved metal tolerance in seedlings by decreasing metal uptake and increasing the levels of metal-binding metallothionein proteins.

## 1. Introduction

Global agriculture is confronting significant challenges in meeting the growing demands for food consumption. Food security largely depends on the increased production of cereal crops, mainly wheat (*Triticum aestivum* L.), rice (*Oryza sativa* L.), and maize (*Zea mays* L.) [1]. Over the past century, industrialization has expanded rapidly, leading to an increased exploitation of the Earth’s natural resources. Toxic heavy metals and metalloids, often released as byproducts or waste from mining and industrial activities, are major contributors to environmental pollution and pose significant risks to human health [2]. The presence of these heavy metals in such activities creates substantial ecological and health hazards for humans.

The roots of plants absorb heavy metals and move them to other regions. The soil bioavailability of metal ions affects plant uptake [3]. ATPases/pumps assist plants in maintaining a negative membrane potential during heavy metal absorption. The root epidermis receives heavy metals through channels or organic molecules attached to metal–ligand complexes [3,4]. By limiting photosynthesis and transpiration, these heavy metals drastically reduce plant growth and production. Protease and amylase enzymes may also be affected, preventing germination and seedling growth [5,6]. Minimal mineral absorption contributes to stunted development and cell turgor pressure alterations. Prolonged heavy metal exposure affects phytohormone levels like abscisic acid. Alterations in ABA levels cause inappropriate stomatal behavior because it regulates them. This may permanently close stomata, limiting gaseous exchange and transpiration [7].

Lead (Pb) is the second most toxic metal, making up 0.002% of the Earth’s crust [2,6]. Lead (Pb) and copper (Cu) can be present in soil and water in various chemical forms, each with different levels of bioavailability and potential for harm to living organisms [2,8,9]. Pb primarily exists as Pb^2^^+^ ions, lead carbonates, sulfates, oxides, and organic complexes. Pb^2^^+^, especially under acidic conditions, has the highest bioavailability and toxicity and can be readily absorbed by plant roots [8,10]. Conversely, less soluble forms of Pb, such as carbonates and oxides, are less likely to be absorbed and are less toxic [11]. Likewise, Cu is found in soil as Cu^2^^+^ ions, hydroxides, oxides, sulfides, and organic complexes. Cu^2^^+^ has the highest bioavailability and toxicity, particularly in acidic or low-organic soils, making it easier for plants to absorb Cu and raising the risk of toxicity. As a result, exposure to Cu can lead to oxidative stress and cellular damage [3,9,12]. Cu toxicity results from its interference with cell division in root tips, which inhibits root growth. Additional factors contributing to toxicity include disruptions to microtubules, leakage of K^+^ ions, accumulation of reactive oxygen species (ROS), distorted chloroplast structure, blockage of the electron transport system, and inhibition of enzyme activities [3,9,13].

Cu has two oxidation states: Cu (I) or Cu^+^, and Cu (II) or Cu^2^^+^. As a nutrition, this transition metal is critical to human and agricultural health [3,14]. Several plant physiological activities need Cu. The micronutrient is essential for photosynthesis, respiration, and plant cell wall lignin production. Copper is essential for enzyme activation and carbohydrate and protein metabolism. Cu is essential for agricultural production since shortage stunts plant development and lowers crop output [3,14]. Cu may be toxic or deficient within a small concentration range [3,15]. Cu is inadequate below 0.3 ppm soil concentrations and hazardous over 10 ppm, especially under stress. Normal soil Cu limits are 20 ppm [13,15]. Due to rapid ROS generation and accumulation, plant tissues with high Cu concentrations experience chlorosis and cytotoxicity [15].

Bioremediation, an innovative and eco-friendly method that utilizes microbes to tackle heavy metal pollution, is strongly recommended for agricultural practices due to its cost-effectiveness, environmental advantages, and suitability for large-scale implementation [16,17]. Microorganisms such as bacteria, fungi, and algae convert heavy metals in the environment into their least toxic forms. Bioremediation employs various mechanisms, including adsorption of heavy metals, biotransformation, and sequestration facilitated by microbial metabolites [17]. Fungal species like *Aspergillus*, *Penicillium*, and *Trichoderma* are recognized for their potential to degrade agricultural organic pollutants, such as pesticides, herbicides, and insecticides, through specialized enzymes, thereby aiding in the remediation of contaminated agricultural fields [18]. Due to their symbiotic relationship with plants, fungal hyphae trap heavy metals in the soil. These symbiotic fungi upgrade plant enzymes, especially acid phosphatase, to improve nutrient intake and development [19,20]. Recent studies have identified and analyzed many heavy metal-adapted rhizofungi. To understand how metal-resistant rhizofungi affect plant development and survival under harsh circumstances, further study is needed. This study intends to establish sustainable agriculture. Thus, phytoremediation may remove heavy metals from polluted environments.

In the present study; we aimed to explore the tolerance threshold of *Aspergillus terreus* for phytoremediation and also its role in Cu uptake under Pb toxicity. We hypothesized that *A. terreus* contributes to Pb detoxification and affects the physiological, biochemical, and biochemical parameters in *T. aestivum* L.

## 2. Results

### 2.1. Rhizospheric Fungus Isolation and Screening Against Heavy Metal Tolerance

**Figure 1:**
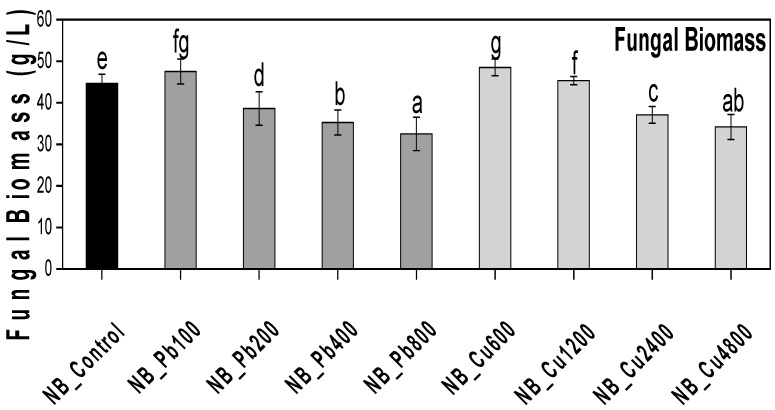
Assessment of the copper and lead tolerance potential of NB (*A. terreus*). The isolate was grown in shaking flasks (250 mL) containing 50 mL Czapek broth amended with different concentrations of Pb and Cu. Mean of triplicated data with standard error and letter labels for denoting significance are given (ANOVA-Duncan *p* < 0.05).

*P. hysterophorus* was selected for isolation, resulting in the recovery of eight fungal strains (NMG, NDB, N6, NW, NP, NB, NYW, NG). These strains were cultured on PDA media in Petri dishes until pure colonies were obtained. Subsequently, the strains were screened on Czapek media against various concentrations of Pb and Cu. The isolate NB (*A. terreus*) demonstrated effective tolerance to both heavy metals, as evidenced by the substantial biomass produced in broth containing these metal concentrations (Figure 1).

### 2.2. Assay for Plant Growth Promoting Potential of the Isolated Fungi

The tolerance of fungal strains isolated from *P. hysterophorus* to heavy metals was evaluated by cultivating them on plates with 75 ppm of Pb and 200 ppm of Cu. The results indicate that four of the eight fungal strains (NMG, NG, N6, and NB) are able to tolerate high concentrations of both metals and shows potential for promoting the growth of *T. aestivum*. Among these four strains, NB (*A. terreus*)-inoculated seedlings exhibited the greatest growth, with root and shoot lengths 94% and 89% greater than the control, respectively. The tolerance of this fungal strain was assessed by measuring the growth of *T. aestivum* in the presence of 75 ppm Pb and 200 ppm Cu. The results show that the fungal strain NB (*A. terreus*) are able to grow in the presence of both metals, suggesting its potential for use in bioremediation efforts.

### 2.3. Characterization of the Selected Isolate

**Figure 2:**
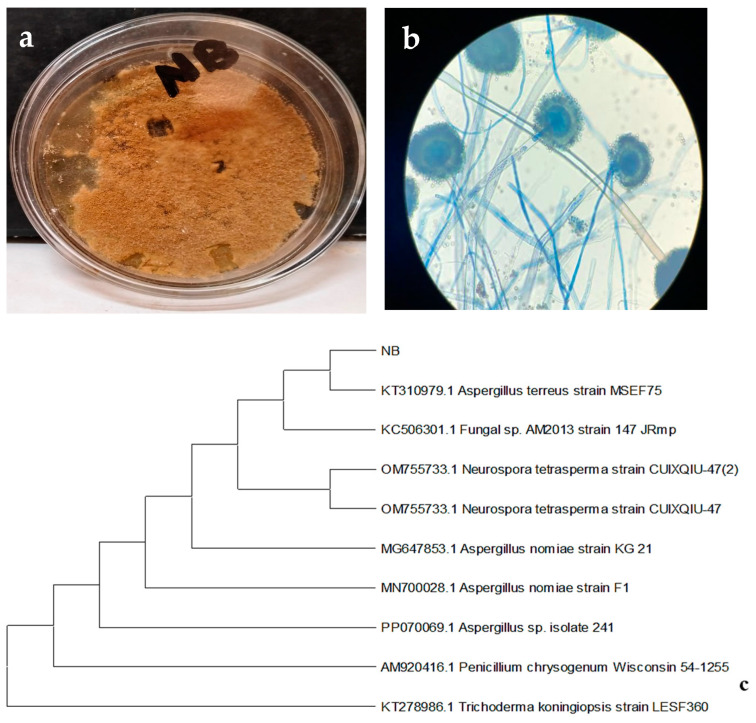
Colony morphologies on (**a**) a macroscopic and (**b**) a microscopic scale of the isolated rhizospheric fungi. (c) Phylogenetic tree based on ITS rDNA sequences of the rhizospheric fungal strain *A. terreus* was constructed.

The rhizofungal isolate NB (*A. terreus*), which promotes wheat seedling growth and tolerates heavy metals (Pb and Cu) in culture media, was selected. This rhizospheric fungus exhibited dark olive-colored colonies with a velvety texture, changing from white to brown shades as the colonies matured. Under a light microscope, conidiophores and conidia were observed, with conidiophores bearing conidia (asexual spores) typically produced in chains. The hyphae of the fungus were aseptate. The fungal strain is identified up to the genus level (Figure 2).

### 2.4. Identification Based on ITS Sequences and Phylogenetic Analysis

To identify the taxonomic status of NB strain, the ITS rDNA sequencing and phylogenetic analysis were performed. The final results of the sequence obtained were compared to the available data in the GenBank sequence database to identify the genus or species (GenBank Accession No. KT310979.1). After searching for homology on GenBank, the sequence of the NB strain was identified up to species, which showed 100% similarity with *A. terreus* (Figure 2).

### 2.5. Effect of Lead Acetate and Copper Sulfate on Fungal Growth

The selected rhizofungus (NB) was tested for its ability to withstand heavy metal (Pb and Cu) stress. The rhizofungus shows exceptional growth in Czapek broth containing varying quantities of Pb and Cu. In a medium containing 100 ppm of Pb, fungal growth was enhanced, as evidenced by a 48.5% increase in biomass compared with the control (medium without Pb). However, higher concentrations of Pb were detrimental to fungal growth, with a dose-dependent decrease in biomass observed. For example, biomass decreased by 61% when exposed to 200 ppm Pb. At Pb concentrations of 400 and 800 ppm, biomass fell to 64% and 67% of the levels observed in the absence of Pb, respectively. Fungal cultures grown in media containing Cu showed similar responses. At 600 ppm Cu concentration, fungal biomass exceeded the control level. However, biomass significantly decreased at 1200 ppm, 2400 ppm, and 4800 ppm. The lowest biomass, recorded at 4800 ppm, is 65%, which is 10% lower than that of the control.

### 2.6. Release of Sugars and Proteins by the Rhizofungus

**Figure 3:**
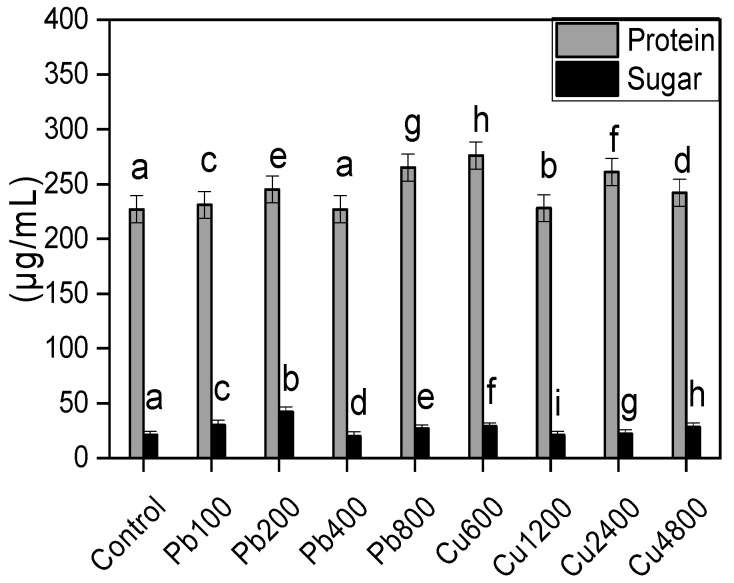
Release of soluble sugars and proteins by NB (*A. terreus*) fungus in response to varied amounts of lead acetate and copper sulfate stress. Data are the means of duplicates with standard error and letter labels denoting significance (ANOVA-Duncan *p* < 0.05).

In general, the seedlings accumulated greater quantities of sugars when grown in soil containing varying concentrations of Pb and Cu. Under Pb stress, the release of sugars increases significantly, peaking at 200 ppm (Figure 3). However, at higher Pb concentrations, the amount of sugar released declined markedly. When exposed to various Cu concentrations, the amount of sugar released was significantly higher compared with control cultures. For instance, the sugar release was 38% greater than the control when the rhizofungus was cultured in a medium containing 600 ppm Cu. The isolate also released higher quantities of protein under stress conditions. There is a gradual increase in protein levels with rising Pb concentrations (Figure 3). In the case of Cu, protein release was more than twice as high in the presence of 600 ppm Cu. Nonetheless, further increases in metal concentration led to a decrease in protein release, although it remained higher compared with control and Pb-stressed cultures.

## 3. Growth of *T. aestivum* under Various Conditions

### 3.1. Shoot Length and Root Length

**Figure 4:**
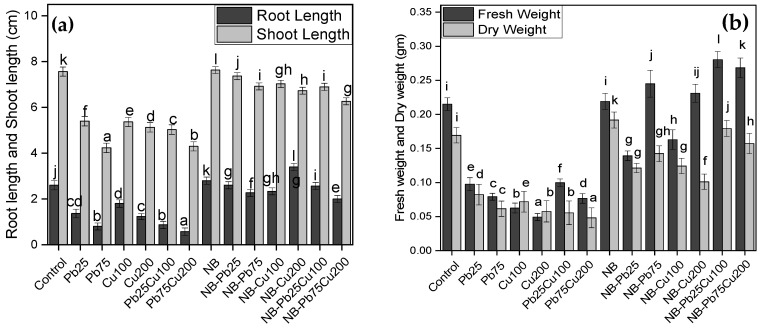
(**a**) Root length, shoot length, (**b**) fresh weight, and dry weight of *T. aestivum* L. in response to varied amounts of lead acetate and copper sulfate stress treated with NB (*A. terreus*). Data are the means of duplicates with standard error (Duncan test; *p* < 0.05).

The shoot and root lengths of *T. aestivum* were measured under various treatments of Pb and Cu, with and without the presence of *A. terreus*. Both heavy metals, Pb and Cu, caused a significant reduction in shoot and root lengths compared with the non-stressed seedlings (Figure 4). For instance, the shoot length of seedlings grown in soil contaminated with 25 ppm and 50 ppm of Pb dropped by 47% and 69% of the non-stressed seedlings, respectively. The same group of seedlings had an average root length dropped by 28% and 44% compared with non-stressed seedlings (Figure 4). Exposure to Cu also had negative effects, causing up to 32% and 52% reductions in shoot and root length compared to non-stressed seedlings. However, seedlings inoculated with the fungus demonstrated improved growth, with root and shoot lengths increased by 30% and 11%, respectively, compared with the control. When fungal-inoculated seedlings were grown in Pb- or Cu-contaminated soils, toxicity was reduced, and growth was enhanced. For instance, the shoot length of seedlings with NB inoculation was greater under Pb stress (25 ppm) compared with non-stressed seedlings. While root growth was lower than that of non-stressed fungal-associated seedlings, it was still superior to the control group. Higher Pb levels (75 ppm) reduced root and shoot lengths in fungus-colonized seedlings, but these measurements remained higher than in the control. Similarly, exposure to Cu had a minimal impact on seedling growth.

### 3.2. Fresh Weight and Dry Weight

Seedlings’ ability to accumulate biomass was also adversely affected under Pb and Cu stress (Figure 4). For Pb, the most significant impact on biomass accumulation occurred in seedlings exposed to 75 ppm, where fresh and dry weights dropped to 63% and 64% of the control levels, respectively. Similarly, the presence of 200 ppm Cu in the soil resulted in the greatest reduction in fresh weight (77%) and dry weight (70%) compared with the control. However, the rhizofungus enhanced biomass accumulation in seedlings, leading to a 7% increase in fresh weight and a 40% increase in dry weight compared with the control. Seedlings occupied by NB showed tolerance to metal exposure, with minimal to negligible impact on their fresh and dry weight.

### 3.3. Chlorophyll a and b

**Figure 5:**
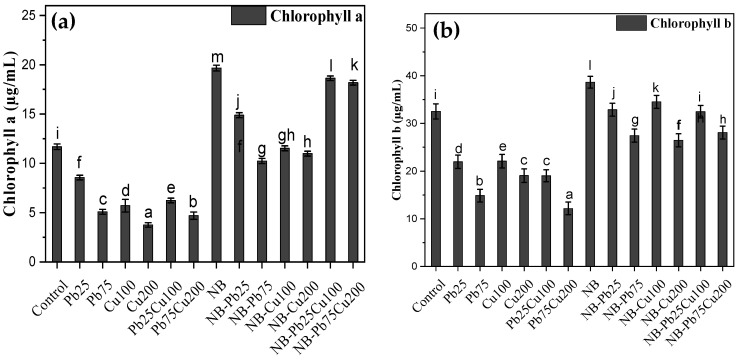
(**a**) Chlorophyll a, and (**b**) chlorophyll b of *T. aestivum* L. in response to varied amounts of lead acetate and copper sulfate stress treated with NB (*A. terreus*). Data are the means of duplicates with standard error (Duncan test; *p* < 0.05).

Metal exposure led to a significant reduction in the level of chlorophyll a and b of the seedlings (Figure 5). For instance, treatment of the seedlings with 75 ppm Pb decreased the levels of these pigments to 44% and 46% of the control group, respectively. Cu treatment also had a significant impact on these pigments, and the presence of 200 ppm Cu led to up to 68% decrease in the level of chlorophyll. Inoculating the seedlings with the rhizofungus *A. terreus*, not only improved chlorophyll contents but also mitigated the impact of heavy metals stress (Figure 5).

### 3.4. SOD, POD, CAT, and Electrolyte Leakage

**Figure 6:**
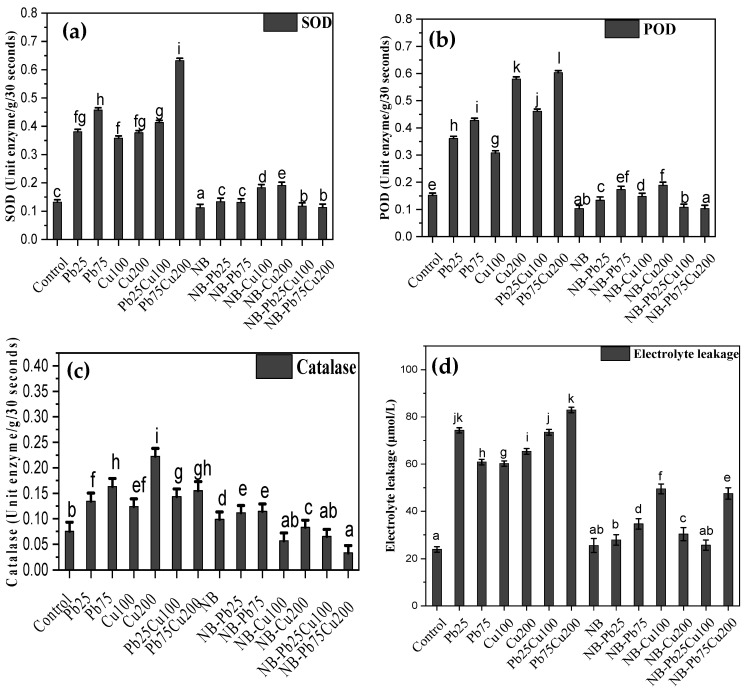
(**a**) SOD, (**b**) POD, (**c**) CAT, and (**d**) electrolyte leakage of *T. aestivum* L. in response to varied amounts of lead acetate and copper sulfate stress treated with NB (*A. terreus*). Data are the means of duplicates with standard error (Duncan test; *p* < 0.05).

Exposure of *T. aestivum* to high levels of Cu and Pb affected the production and accumulation of various enzymes, including SOD, POD, and CAT (Figure 6). With Pb treatment, the quantity of SOD was 79% higher at 25 ppm and 117% higher at 75 ppm compared with the control. Cu treatment also led to increased accumulation of SOD in seedlings. The combined effect of Pb and Cu resulted in a synergistic increase, producing the highest levels of SOD observed in this study. Levels of POD and CAT were also elevated in metal-treated seedlings, with increases directly related to metal concentrations. For instance, 75 ppm Pb induced an 181% increase in POD levels compared with the control, while 200 ppm Cu raised POD concentration by up to 282% compared with control seedlings. When combined, 75 ppm Pb and 200 ppm Cu resulted in a 296% increase in POD activity compared with the control. In contrast, inoculation with the NB fungus reduced SOD and POD activities, although CAT levels were increased by 31% in the presence of NB fungus, though this was still less than the metal-induced increase. When metal treatments were applied to seedlings associated with NB fungus, the increase in enzyme accumulation was significantly reduced (Figure 6).

Exposure to metals also caused the membranes of seedlings to become more porous, leading to increased electrolyte leakage compared with the control (Figure 6). For example, seedlings treated with 25 ppm and 75 ppm Pb showed 211% and 155% greater electrolyte leakage, respectively, compared with the control. Cu treatment similarly increased electrolyte leakage by 152% (100 ppm) and 174% (200 ppm) compared with the control. The combined presence of these metals had a synergistic effect, further exacerbating membrane damage and electrolyte leakage. In contrast, NB fungus did not affect electrolyte leakage, as there was no significant difference between the control and fungus-associated seedlings. Additionally, the impact of metals and their combinations is significantly reduced in seedlings associated with the fungus (Figure 6).

### 3.5. H_2_O_2_ and MDA Content

**Figure 7:**
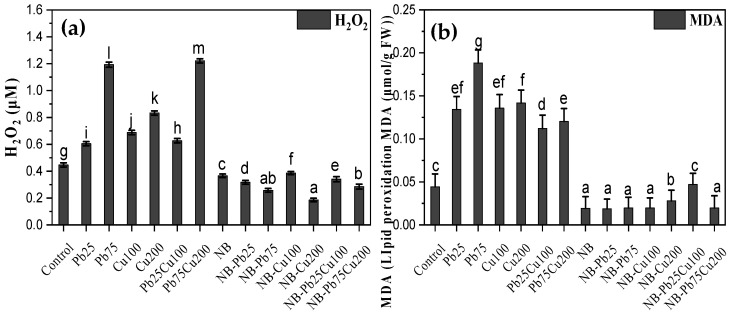
(**a**) H_2_O_2_ and (**b**) MDA content assessment of *T. aestivum* L. in response to varied amounts of lead acetate and copper sulfate stress treated with NB (*A. terreus*). Data are the means of duplicates with standard error (Duncan test; *p* < 0.05).

Besides elevated levels of antioxidant enzymes, accumulation of H_2_O_2_ was still higher in heavy metal-treated seedlings than in the control (Figure 7). For instance, treatment of seedlings with 25 and 75 ppm of Pb led to 35% and 167% higher values of endogenous H_2_O_2_ compared with the control. Cu treatment also resulted in increased H_2_O_2_ production, with 54% and 86% higher accumulation compared with the control. The combined application of Pb and Cu further promoted ROS accumulation. This was accompanied by elevated levels of malondialdehyde (MDA) in seedlings treated with heavy metals. However, seedlings inoculated with the rhizofungus NB showed lower levels of H_2_O_2_ and MDA compared with the control. Treatment of rhizofungus-colonized seedlings with Pb, Cu, or their combination did not significantly affect the accumulation of these stress markers (Figure 7).

### 3.6. Sugar and Protein Content in Triticum aestivum L.

**Figure 8:**
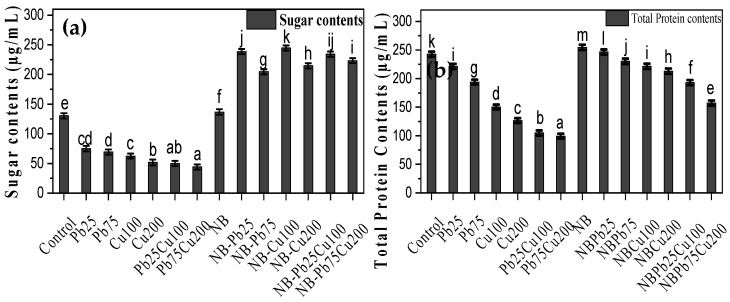
(**a**) Total protein, and (**b**) sugar content assessment of *T. aestivum* L. in response to varied amounts of lead acetate and copper sulfate stress treated with NB (*A. terreus*). Data are the means of duplicates with standard error (Duncan test; *p* < 0.05).

Pb and Cu negatively affected the protein and sugar content of seedlings (Figure 8). Specifically, Pb treatment resulted in the lowest levels of sugars at 75 ppm, reducing its concentration to 50% of the control plants. *T. aestivum* L. grown in media with 200 ppm Cu had 60% less sugar compared with the control. In contrast, the rhizofungus *A. terreus* enhanced the sugar content in wheat seedlings, causing a 71% increase in sugar concentration. Similarly, seedlings associated with *A. terreus* maintained higher sugar levels even in the presence of Pb and Cu. The total protein content in *T. aestivum* L. exposed to 25 pp of lead and 100 ppm of copper was significantly reduced, showing a decrease of 56% compared with that in plants subjected to the same metal stress but treated with rhizofungus.

### 3.7. Rhizofungal Colonization in Roots of T. aestivum L.

**Figure 9:**
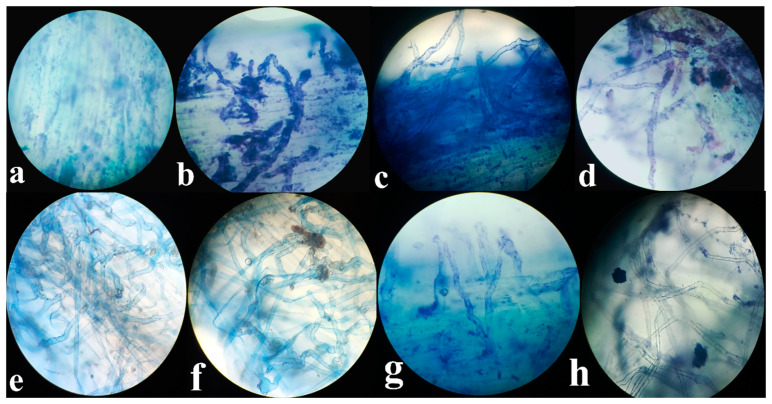
Colonization of the rhizofungus NB (*A. terreus*) in the roots of *T. aestivum* L. exposed to different concentrations of Pb and Cu; (**a**) control, (**b**) NB (*A. terreus*), (**c**) NB + Pb 25 ppm, (**d**) NB + Pb 75 ppm, (**e**) NB + Cu 100 ppm, (**f**) NB + Cu 200 ppm, (**g**) NB + Pb 25Cu100 ppm, (**h**) NB + Pb75Cu200 ppm. Root sections were stained with lactophenol cotton blue and observed under a light microscope at 40× magnification.

Root colonization of isolated rhizospheric fungi in *T. aestivum* was observed microscopically using lactophenol (cotton blue) following exposure to copper and lead stress (Figure 9). The results indicate that *A. terreus* effectively colonizes the root tissues of *T. aestivum* seedlings. Additionally, the presence of heavy metals (Pb and Cu) significantly increases the amount of colonized hyphae.

### 3.8. Expression of Phytochelatin and Metallothionein Genes under Heavy Metal Stress

**Figure 10:**
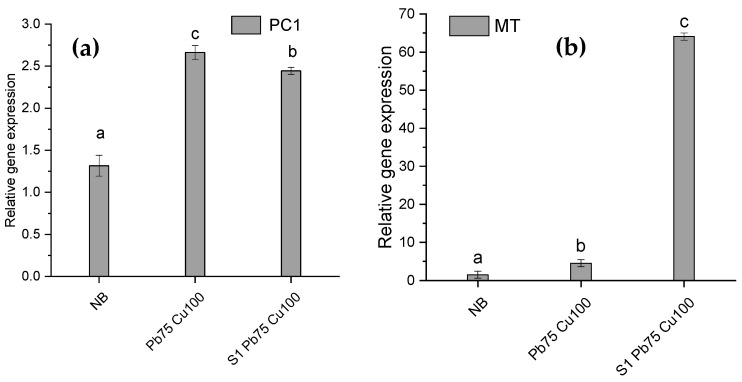
Role of NB (*A. terreus*) with lead acetate [Pb(C_2_H_3_O_2_)_2_] and copper sulfate [CuSO_4_] on (**a**) phytochelatin (PC1) and (**b**) metallothionein (MT1) gene expression in *T. aestivum* L. cultivated in soil polluted with Pb and Cu. Data are the means of duplicates with standard error (Duncan test; *p* < 0.05).

*T. aestivum* L. plants were grown under both control and stress conditions with rhizofungus treatment to evaluate the expression of phytochelatin and metallothionein genes. The expression levels of these genes in various *T. aestivum* L. groups are shown in Figure 10. It was observed that fungal inoculation resulted in a 2.44-fold increase in phytochelatin gene expression and a 64-fold increase in metallothionein gene expression (Figure 10). Exposure to the combined metals Cu and Pb led to a 2.66-fold increase in phytochelatin gene expression and a 4.5-fold increase in metallothionein gene expression. Compared with non-NB seedlings, the expression of the phytochelatin gene was lower in NB-associated seedlings. Conversely, metallothionein gene expression was significantly higher in NB-colonized seedlings compared with non-NB seedlings when exposed to the Cu and Pb combination (Figure 10).

### 3.9. Scanning Electron Microscopy of Triticum aestivum L. Roots

**Figure 11:**
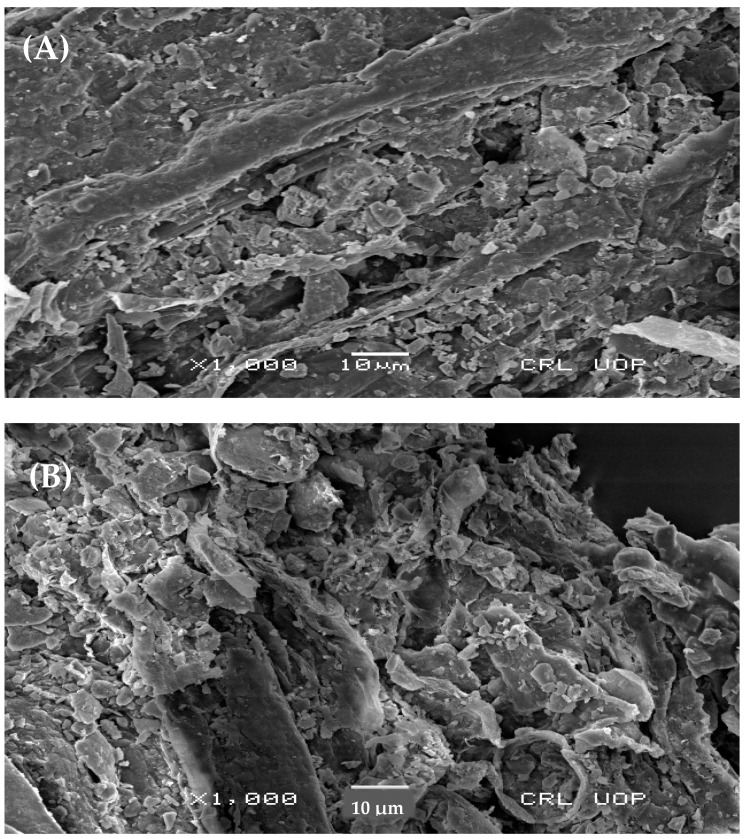

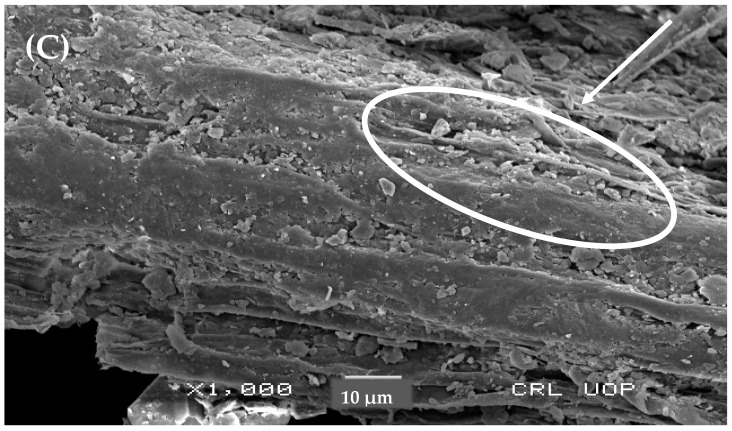
The scanning electron microscopy (SEM) picture of the root surface show the presence of strain *A. terreus* on it. (**A**) The root of *T. aestivum* plant without inoculation. (**B**) The fine structure of *T. aestivum* roots, subjected to both induced Pb and Cu stresses at the minutest level. (**C**) Hyphae of the *A. terreus* strain (the white arrows show the position). This activity was performed in Centralized Resource Laboratory (CRL), University of Peshawar.

*T. aestivum* plants inoculated with *A. terreus* under induced Pb and Cu stress were analyzed using SEM. The results revealed extensive colonization of rhizofungus hyphae in the plant roots. The control samples exhibited normal morphology with no fungal colonization (Figure 11A), while samples exposed to Pb and Cu stress showed disrupted morphology, highlighting the severe impact of these metals on plants (Figure 11B). In contrast, *A. terreus*-colonized *T. aestivum* L. seedlings under Pb and Cu stress maintained normal cell structure (Figure 11C), demonstrating the positive role of the fungus in mitigating toxic metal stress in plants.

### 3.10. Energy Dispersion Spectroscopy

Energy dispersive X-ray analysis of *T. aestivum* L. plants subjected to Pb and Cu stress, along with rhizospheric fungi, revealed insights into elemental composition and metal uptake mechanisms (Figure S1). The results indicate that no heavy metal accumulation occurs in roots grown without these metals. However, exposure to a combination of Pb and Cu led to their accumulation in root tissues. This heavy metal exposure also disrupted the uptake and accumulation of other nutrients, particularly chlorine and silicon. Fungal inoculation effectively reduced Pb uptake and accumulation in roots grown in soil containing both Cu and Pb and restored phosphorus levels to normal. However, fungal inoculation did affect Cu uptake and accumulation in the root tissues. The potential of rhizospheric fungus to enhance Cu accumulation in root tissues under Pb stress was evident. The isolated fungus not only promoted copper uptake but also facilitated the absorption of essential elements like magnesium, potassium, iron, and calcium under toxic heavy metal stress compared with the control (Figure S1).

## 4. Discussion

This study investigates the individual and combined impacts of the heavy metals Pb and Cu on the growth and metabolism of *T. aestivum* seedlings and evaluates the potential of the rhizofungus *A. terreus* for managing these stresses. To achieve this, this study first involved isolating rhizofungi from *P. hysterophorus* plants growing near an industrial site in the Mardan District. These isolates were subsequently subjected to a two-stage screening process to assess their tolerance to heavy metals and their potential for plant growth promotion. Based on the screening assay, the strain NB was selected and identified as *A. terreus* using the internally transcribed spacer (ITS) region of its rDNA. This strain, isolated from the rhizosphere of *P. hysterophorus*, is known for various ecological functions, including antagonistic activity against bean root rot, the production of cytotoxic metabolites, and antiviral properties [21,22,23]. Exposure to low concentrations of heavy metals improves fungal growth, but increasing concentrations of Pb to 200 ppm and above and Cu to 400 ppm and above reduces fungal growth. These findings suggest that the selected isolate can tolerate both fungi as their concentration in soil or water mostly remains lower than 100 ppm [24].

The rhizofungus *A. terreus* not only grows well but also releases metabolites related to stress tolerance in fungi and host plants. It released greater quantities of soluble sugars when grown in Pb- and Cu-supplemented media. Soluble sugars not only help organisms sense their environment but also modulate the expression of stress-responsive genes [24,25]. They can be absorbed by the host plants to compensate for the deficit in soluble sugars induced by heavy metal stress [25]. The fungus’ enhanced protein production in response to heavy metals is beneficial because metal-binding proteins alter plant roots’ metal absorption. Additionally, extracellular proteins biosorb metal [9,20,26], improving fungal metal tolerance and perhaps lowering plant root metal availability.

The ability of the rhizofungus to tolerate heavy metals and release stress-related metabolites enabled it to mitigate these heavy metals in the host seedlings, restoring their growth to normal in the soil contaminated with Pb and Cu [24]. Growth restoration was accompanied by improved chlorophyll contents in the leaves, reduced by the stress in the absence of fungal inoculation [5]. Microbial inoculations are known to improve chlorophyll contents, and several factors may be responsible for this increase, such as reduced metal uptake, chelation of heavy metals and improved antioxidant defense system [4,26,27]. In fact, we noticed a higher accumulation of ROS in metals-exposed seedlings, which was brought to normal levels in fungus-inoculated seedlings [9]. Metal exposure can disturb the balance of ROS generation and their scavenging by affecting metabolism to drive the generation of higher levels of ROS, overburdening the antioxidant system [28]. However, fungal inoculation can reduce metal exposure, suppressing ROS generation, and improving the antioxidant system to keep ROS levels under control [27]. In *A. terreus*-colonized seedlings, SOD and POD activities were lower than in controls, while catalase activity was higher. Antioxidant enzyme levels increased under heavy metal exposure but remained lower than in inoculated seedlings grown in contaminated soil. This balance effectively maintained normal H_2_O_2_ levels, with *A. terreus* modulating metal-induced changes in these enzymes [9,19,29].

Excess ROS leads to lipid peroxidation, measured by MDA levels, causing membrane damage, electrolyte leakage, and impaired plant growth [9,28,30]. Our data show that excess ROS in Cu and Pb-stressed wheat seedlings correlated with increased MDA levels and greater electrolyte leakage. ROS activates ion channels, causing K^+^ leakage, which can trigger cell death or support stress adaptation [9,28]. Excess ROS, MDA, and electrolyte leakage collectively hindered growth and pigment accumulation in stressed seedlings. However, in *A. terreus*-colonized seedlings, ROS levels were controlled, keeping MDA and electrolyte leakage within normal ranges.

To understand how the fungus helps plants adapt to stress, we examined wheat seedlings’ phytochelatin (PC1) and metallothionein (MT) gene expression under different treatments. We used SEM and energy-dispersive X-ray spectroscopy to measure heavy metal intake and buildup. Plants need phytochelatins and metallothioneins to detoxify and tolerate heavy metals [26]. Both fungal inoculation and metal exposure significantly elevated PC1 and MT expression. An interesting finding was up to 64-fold higher expression of the MT gene in metal-exposed rhizofungus-associated seedlings. These cysteine-rich small peptides are known for their stronger interaction with Cu, limiting its access to the living tissues. This is in line with the SEM-EDS data, which showed that fungal inoculation did not affect Cu uptake by the host roots. Hence, the excess of Cu was chelated by MTs, abundantly produced and accumulated in such seedlings [26]. MT’s biological functions include heavy metal detoxification, oxidative defense, and homeostasis. It detoxifies heavy metals by complexing with them and prevents them from interacting with vital macromolecules. Although not to the same extent, phytochelatins genes were also upregulated in the presence of HMs and rhizofungus inoculation. Phytochelatins detoxify heavy metals by binding to and sequestering them in plant vacuoles, reducing their harmful effects on plant tissues and limiting their translocation to the above-ground parts [26]. Our findings indicate that wheat seedlings use PC1 and MT to minimize heavy metal toxicity, with *A. terreus* improving tolerance via MT gene expression.

## 5. Materials and Methods

### 5.1. Isolation and Culturing of Rhizofungal Strains

Parthenium plants were collected from selected sites in Mardan District (34°12′0 N 72°1′60 E) in the Khyber Pakhtunkhwa province of Pakistan by digging them up with a spade, along with the surrounding soil. The roots were vigorously shaken in the air to remove excess soil. The rhizosphere soil was then collected by shaking the roots in a 1 L beaker filled with autoclaved distilled water. A serial dilution method was then employed to isolate the soil-borne fungi residing in the rhizosphere [21], with dilutions ranging from 10^−2^ to 10^−5^. To prevent bacterial growth, 20 mL of potato dextrose agar (PDA) supplemented with streptomycin (2.5 mg/mL) was aseptically poured into sterile Petri dishes. A 100 µL aliquot from each dilution was evenly spread across the surface of the agar using a spreader. After sealing the plates with Scotch tape, they were incubated at 28 °C for three to seven days. The fungal colonies, carefully removed from their Petri dishes, were transferred to freshly prepared PDA slants and PDA plates. These were then labeled accordingly and incubated for seven days at 28 ± 2 °C. The pure culture PDA slants were stored at 4 °C for future use.

### 5.2. Assessment of Fungal Isolates for Cu and Pb Tolerance

The fungal isolates were cultivated in Czapek media (Sigma-Aldrich, American chemical, life science, and biotechnology company) and exposed to various concentrations of Pb (100 µg, 200 µg, 400 µg, and 800 µg) and Cu (600 µg, 1200 µg, 2400 µg, and 4800 µg) to assess their ability to withstand these metals. The cultures were shaken for 5–7 days under the conditions mentioned above. After completion of the incubation period, fungal biomass was filtered and weighed. Lead acetate and copper chloride were used as sources of Pb and Cu, respectively.

### 5.3. Molecular Identification of Rhizospheric Fungal Isolate

DNA was extracted from freeze-dried mycelium for molecular identification. Extraction of DNA was performed by the phenol/chloroform method [31]. Amplification and sequencing of the fungal ITS 1 and 4 regions of rDNA were performed by standard protocol [32]. Both forward and reverse reads were aligned to reconstruct the final consensus sequence, which was compared to closely matching sequences in GenBank using NCBI BLAST (https://blast.ncbi.nlm.nih.gov/Blast.cgi, accessed on 17 June 2024). The close hits were used to assess the phylogenetic relationship of the ITS sequence of our isolate through MEGA-11 [33].

### 5.4. Experimental Design of Plant–Microbe Interaction Experiment

This study was conducted in a naturally lit greenhouse at Abdul Wali Khan University, Mardan, Pakistan, from 4 November to 3 January. The temperature ranged from 20 to 25 °C, with relative humidity fluctuating and averaging 66.5%. The duration of daylight and darkness was 11 h and 13 h, respectively. Before starting the experiment, soil samples were collected from the AWKUM agricultural field, and the selected physicochemical properties were analyzed. The soil was sandy loam with a pH of 7.81, an electrical conductivity (EC) of 277 μmhos cm^−1^, and concentrations of nitrogen, phosphorus (P), and potassium at 95.86, 8.62, and 109.35 mg kg^−1^, respectively. The total amount of lead extractable with DTPA (diethylenetriaminepentaacetic acid) was 2.31 mg kg^−1^. Lead acetate [Pb(C_2_H_3_O_2_)_2_] was dissolved in double-distilled water (DDW) to prepare a lead stock solution with a concentration of 10 g/L. The seeds of *T. aestivum* (Peer sabaq) were surface-sterilized using a 0.01% mercuric-chloride solution and then placed in plastic pots (autoclaved) with a 3:1 seed-to-pot ratio. All treatments were replicated three times and arranged in a fully randomized block design (Table 1). The soil was amended with copper chloride at concentrations of 100 µg/mL and 200 µg/mL, rhizospheric fungus (NB), and lead acetate at concentrations of 25 µg/mL and 75 µg/mL as sources of Pb. The pots were irrigated with distilled water as needed to ensure consistent and controlled conditions throughout the experiment. The treatments included:

The concentrations of Cu and Pb were chosen from the first screening tests. Lead acetate [Pb(C_2_H_3_O_2_)_2_] and copper sulfate [CuSO_4_] were added to the soil before seeds were sown. The experiment involved the use of analytical grade substances to assess the impact of heavy metals on plant growth and biochemical characteristics. Plants were regularly irrigated and were harvested after sixty days of sowing to evaluate various growth and biochemical parameters. This study aimed to understand how different heavy metal concentrations affect plant development and physiological responses.

### 5.5. Growth Parameters of Wheat Seedlings

Seedlings growth was estimated by measuring the length (shoot and root lengths) and biomass (Fresh and dry weight) of the harvested seedlings. For estimation of pigments, fresh leaves were extracted in 80% acetone, and the extract was assessed for absorbance at 663 nm and 645 nm using a UV–Vis spectrophotometer (T70, United Kingdom, UK). For pigments estimation, ref. [14] was used as follows:

$$Chlorophyll\;a\;({µgmL}^{-1})\;=\;(12.25\;A663\;-\;2.79\;A647)$$

$$Chlorophyll\;b\;({µgmL}^{-1})=(21.50\;A647-5.14\;A663)$$

$$Total\;Chlorophyll\;({µgmL}^{-1})=Chlorophyll\;a+Chlorophyll\;b$$

$$\mathrm{Carotenoids}(µ{\mathrm{gmL}}^{-1})\;\;\frac{\left(1000\;A470-1.82\;Chlorophyll\;a-85.02\;Chlorophyll\;b\right)}{198}$$

where *A*663 is the absorbance at 663 nm, and *A*647 is the absorbance at 647.

### 5.6. Estimation of Antioxidant Enzymes

Extraction and determination of the antioxidant enzymes from the seedlings were performed as described previously [34]. Enzyme extraction and quantitation were carried out as described previously. Briefly, 1 g of frozen leaves of the seedlings were extracted with phosphate buffer (pH 7), and the supernatant was obtained by carrying out centrifugation at 12,000× *g* for 15 min and 4 °C. The riboflavin/nitroblue tetrazolium (NBT) method was used for the estimation of super oxide dismutase (SOD) activity and for measuring the resultant product at 560 nm. The activity of peroxidase (POD) was determined through the o-dianisidine method, monitoring OD of the product at 470 nm. The activity of catalase (CAT) was determined by using H_2_O_2_ as a subtract. Activities of all these enzymes were expressed in micrograms per milligram of protein.

### 5.7. Electrolyte Leakage

To estimate the leakage of electrolytes, leaf discs were treated with dH_2_O, and electrical conductivity was recorded (EC1) with the help of an EC meter [35]. After heat treatment, a second reading (EC2) was recorded. The final value was calculated in percentage using the given formula:

$$\mathrm{EC}\;=\;\frac{EC1}{EC2}\;\times 100$$

where EC is the electrical conductivity, EC1 is the initial electrical conductivity, and EC2 is the second electrical conductivity.

### 5.8. H_2_O_2_ Determination

The standard protocol of Velikova et al. [36] was used to extract the hydrogen peroxide from the *T. aestivum* L. seedlings. The concentration of hydrogen peroxide concentration was determined in the extract by measuring absorbance at 390 nm. Potassium phosphate buffer and potassium iodide were used as a blank.

### 5.9. The Malonaldehyde Content Analysis

The MDA content was determined using the TBA (thiobarbituric acid) reaction, following the method described by [37]. Frozen samples were homogenized in 0.1% (*w*/*v*) TCA using a pre-chilled mortar and pestle, followed by centrifugation at 15,000 ×g for 15 min. The resulting supernatant (1 mL) was mixed with 2 mL of 0.5% (*w*/*v*) TBA in 20% (*w*/*v*) TCA and heated at 95 °C for 30 min. The reaction mixture was then rapidly cooled in an ice bath. After final centrifugation at 10,000× *g* for 10 min at 4 °C, the absorbance of the supernatant was measured at 532 nm, and the values were corrected for nonspecific absorption by subtracting the absorbance at 600 nm.

$$MDA\;Conc.\;({\mu mol\;L}^{-1})=6.45\times (A532-A600)-0.56\times A450$$

where MDA Conc. = concentration of malondialdehyde measured in micromoles per liter.

### 5.10. Sugar and Protein Content

The total sugar was determined using the method described by [38] The absorbance was measured at 620 nm using a spectrophotometer. The protein concentration was determined via the Bradford technique [39]. Fresh leaves (0.5 g) were homogenized in 1 mL of phosphate buffer at pH 7.0. The crude homogenate was centrifuged at 5000 rpm for 10 min. Half a milliliter of newly prepared trichloroacetic acid (TCA) was included, and the mixture was centrifuged for 15 min at 8000× *g*. One milliliter of 0.1 N NaOH was used to dissolve the sample, followed by the addition of five milliliters of Bradford reagent. A spectrophotometer (SHIMADZU UV-2450, Kyoto, Japan) was used to quantify the absorbance at 595 nm.

### 5.11. Root Colonization

Fungal hyphae were detected within the tissues of wheat seedlings by using lactophenol cotton blue-stained transverse section of the roots [40]. The stained sections were observed under a light microscope.

### 5.12. Amplification of MT1 and PCS1 Genes

The *T. aestivum* leaves were used to isolate the total cellular RNA extraction. The CDS of MT1 and PCS1 were amplified by PCR using specific gene primers MT1-F (5′-ACACCAAGGGCAGAGCATAG-3′) and MT1-R (5′-CACTCGTGTGATGGTGTGAG–3′).

However, in case of PCS1, we used gene primers PCS1-F 5′CTACTGATAAGG CATTGTTGGAT-3′ and PCS1-R (5′-TGATCC GTGGTGTGAAGC-3′).

### 5.13. Expression Analysis of MT1 and PCS1 in T. aestivum L. under Lead and Copper Stress Condition

*T. aestivum* plants, aged two weeks, were exposed to different concentrations of lead and copper for an additional two weeks before being harvested for RNA extraction using the TRIZOL reagent (Invitrogen, Carlsbad, California) following the manufacturer’s protocol. The actin gene was used as an internal control, with gene-specific primers Actin-F (5′-GTCGGTGAAGGGGACTTACA–3′) and Actin-R (5′-TTCATACAGCAGGCAAGCAC–3′).

### 5.14. Scanning Electron Microscopy

Root extracts from *T. aestivum* plants were air-dried at 35 °C using a vacuum dryer. A small droplet of the dried extract was applied to a carbon-coated SEM grid and allowed to dry completely. The samples were then coated with a thin layer of silver using a spi-module sputter coater (Auto Fine Coater). Morphological features were examined with scanning electron microscopy using an FE-SEM (JSM-5910-JEOL) (Lab Tech, Yokosuka, Japan) at the University of Peshawar.

### 5.15. Energy-Dispersion Spectroscopy (EDS)

Energy-dispersive spectroscopy (EDS) was employed to confirm the presence of Pb and Cu present in the roots of *T. aestivum* L. The sample preparation involved centrifuging the leaf extract at 14,800× *g* for 15 min, followed by drying at 35 °C using a vacuum drier. The resulting dry powder was analyzed using energy-dispersion spectroscopy with a thermal EDS instrument attached to an Oxford Inca 200 SEM (Lab tech., Yokosuka, Japan) at the University of Peshawar.

### 5.16. Statistical Analysis

The replicated data were analyzed using a one-way analysis of variance (ANOVA) with the SPSS 16.0 software. If significant differences were found, pairwise comparisons were performed with Duncan’s multiple range test (DMRT) at *p* = 0.05 to determine which pairs of means were significantly different.

## 6. Conclusions

Current research has demonstrated that 25 mg/kg of Pb and 100 mg/kg of Cu exert toxic effects on plant development, significantly reducing plant biomass. However, inoculating *T. aestivum* seeds with *A. terreus* effectively mitigated Pb and Cu toxicity across various plant parameters. In the treatment group with Pb at 25 mg/kg and Cu at 100 mg/kg, where *A. terreus* was applied, the growth of the seedlings was superior to those in the soil without *A. terreus*. Furthermore, Cu at 200 mg/kg reduced plant defense enzymes (SOD, POD), as well as sugar content in Cu-contaminated soil, but these levels increased with *A. terreus* treatment. In addition, inoculation with *A. terreus* demonstrated the ability to confer resistance against both lead and Cu stresses, enhancing the levels of POD and SOD enzymes in *T. aestivum*. Fungus associated seedlings had significantly lower levels of Pb in the roots compared with the non-inoculated seedlings. The expression of PCs and MTs genes was observed under Pb and Cu stress, which helps bind heavy metals, reducing their toxicity and facilitating their sequestration and removal from plants. Inoculation with *A. terreus* helped *T. aestivum* plants effectively restore cell structure under Pb and Cu stress. Therefore, *A. terreus* could be recommended as a plant growth-promoting rhizofungal inoculum for enhancing plant growth in heavy metal-polluted conditions and could serve as a biofertilizer to stimulate plant development.

## Figures and Tables

**Table 1:** Shows the treatments and fungal inoculation under stressful conditions.

Condition	Treatments	µg/mL
Normal condition	Control	Untreated
Copper and lead stress	Pb25	25 µg/mL
	Pb75	75 µg/mL
	Cu100	100 µg/mL
	Cu200	200 µg/mL
	Pb25 + Cu100	25 µg/mL + 100 µg/mL
	Pb75 + Cu200	75 µg/mL + 200 µg/mL
Fungal inoculation	NB (Rhizofungus)	5 g
	NB + Pb25	25 µg/mL
	NB + Pb75	75 µg/mL
	NB + Cu100	100 µg/mL
	NB + Cu200	200 µg/mL
	NB + Pb25 + Cu100	25 µg/mL + 100 µg/mL
	NB + Pb75 + Cu200	75 µg/mL + 200 µg/mL

## Data Availability

The original contributions presented in the study are included in the article; further inquiries can be directed to the corresponding authors.

## Supplementary Materials

The following supporting information can be downloaded at: https://www.mdpi.com/article/10.3390/plants13182643/s1, Figure S1. The energy dispersive x-ray (EDX) image of the root tissue shows the composition of elements, in absence and presence of rhizo-fungus colonization. (A) EDX of non-inoculated root of a *T. aestivum* plant. (B) EDX of the root section of T. aestivum plant under induced Pb and Cu stress. (C) EDX of root sample, inoculated with rhizo-fungus.
