# Correction: Dilawar et al. Rhizofungus *Aspergillus terreus* Mitigates Heavy Metal Stress-Associated Damage in *Triticum aestivum* L. *Plants* 2024, *13*, 2643

**DOI:** 10.3390/plants15132039

**Published:** 2026-07-01

**Authors:** Naveen Dilawar, Muhammad Hamayun, Amjad Iqbal, Bokyung Lee, Sajid Ali, Ayaz Ahmad, Abdulwahed Fahad Alrefaei, Turki Kh. Faraj, Ho-Youn Kim, Anwar Hussain

**Affiliations:** 1 Department of Botany, Abdul Wali Khan University Mardan, Mardan 23200, Pakistan; naveen@awkum.edu.pk (N.D.); hamayun@awkum.edu.pk (M.H.); 2 Department of Food Science and Technology, Abdul Wali Khan University Mardan, Mardan 23200, Pakistan; amjadiqbal@awkum.edu.pk; 3 Department of Food Science and Nutrition, Dong-A University, Busan 602760, Republic of Korea; bolee@dau.ac.kr; 4 Department of Horticulture and Life Science, Yeungnam University, Gyeongsan 38541, Republic of Korea; 5 Department of Biotechnology, Abdul Wali Khan University Mardan, Mardan 23200, Pakistan; ahdayazb5@awkum.edu.pk; 6 Department of Zoology, College of Science, King Saud University, Riyadh 2455, Saudi Arabia; afrefaei@ksu.edu.sa; 7 Department of Soil Science, College of Food and Agriculture Sciences, King Saud University, Riyadh 2455, Saudi Arabia; talasiri@ksu.edu.sa; 8 Korea Institute of Science and Technology, Gangneung 25451, Republic of Korea; hykim@kist.re.kr

In the original publication [1], the affiliation of author Ho-Youn Kim was incorrectly listed as “Korean Institute of Science and Technology”. This has been corrected to “Korea Institute of Science and Technology”.

In the original publication [1], several references cited in the manuscript did not appropriately support the statements they were associated with. Upon careful re-examination, the authors identified that the reference list contained numerous inaccuracies, including irrelevant citations, duplicated entries, and mismatched reference–statement pairings. The entire reference list has been thoroughly revised and corrected as below:

1. UNICEF. *The State of Food Security and Nutrition in the World 2023*; UNICEF: New York, NY, USA, 2023.
2. Nagajyoti, P.C.; Lee, K.D.; Sreekanth, T.V.M. Heavy metals, occurrence and toxicity for plants: A review. *Environ. Chem. Lett.* **2010**, *8*, 199–216.
3. Zhang, M.; Wang, J. Copper bioavailability, uptake, toxicity, and tolerance in plants: A comprehensive review. *Sci. Total Environ.* **2019**, *658*, 618–629.
4. Hall, J.Á. Cellular mechanisms for heavy metal detoxification and tolerance. *J. Exp. Bot.* **2002**, *53*, 1–11.
5. Chandra, R.; Kang, H. Mixed heavy metal stress on photosynthesis, transpiration rate, and chlorophyll content in poplar hybrids. *For. Sci. Technol.* **2015**, *12*, 55–61.
6. Sharma, P.; Dubey, R.S. Lead toxicity in plants. *Braz. J. Plant Physiol.* **2005**, *17*, 35–52.
7. Guo, Z.; Gao, Y.; Yuan, X.; Yuan, M.; Huang, L.; Wang, S.; Liu, C.E.; Duan, C. Effects of heavy metals on stomata in plants: A review. *Int. J. Mol. Sci.* **2023**, *24*, 9302.
8. Waterlot, C.; Pruvot, C.; Marot, F.; Douay, F. Impact of a phosphate amendment on the environmental availability and phytoavailability of Cd and Pb in moderately and highly carbonated kitchen garden soils. *Pedosphere* **2017**, *27*, 588–605.
9. Pandey, N.; Pathak, G.C.; Pandey, D.K.; Pandey, R. Heavy metals, Co, Ni, Cu, Zn and Cd, produce oxidative damage and evoke differential antioxidant responses in spinach. *Braz. J. Plant Physiol.* **2009**, *21*, 103–111.
10. Rahman, S.U.; Qin, A.; Zain, M.; Mushtaq, Z.; Mehmood, F.; Riaz, L.; Naveed, S.; Ansari, M.J.; Saeed, M.; Ahmad, I.; et al. Pb uptake, accumulation, and translocation in plants: Plant physiological, biochemical, and molecular response: A review. *Heliyon* **2024**, *10*, e27724.
11. Porter, S.K.; Scheckel, K.G.; Impellitteri, C.A.; Ryan, J.A. Toxic metals in the environment: Thermodynamic considerations for possible immobilization strategies for Pb, Cd, As, and Hg. *Crit. Rev. Environ. Sci. Technol.* **2004**, *34*, 495–604.
12. Koren’kov, V.D.; Bylina, N.N. Copper in soils and its bioavailability. *Environ. Sci. Pollut. Res.* **2009**, *16*, 579–585.
13. Juang, K.W.; Lo, Y.C.; Chen, T.H.; Chen, B.C. Effects of copper on root morphology, cations accumulation, and oxidative stress of grapevine seedlings. *Bull. Environ. Contam. Toxicol.* **2019**, *102*, 873–879.
14. Arnon, D.I. Copper enzymes in isolated chloroplasts. Polyphenoloxidase in *Beta vulgaris*. *Plant Physiol.* **1949**, *24*, 1–15.
15. Hassan, W.; Zahra, Q.A.; Attia, K.A.; Bashir, S.; Fiaz, S.; Mohammed, A.A.; Mohy-Ud-Din, W.; Aslam, Z.; Hafez, Y.M.; Chen, Z. Assessing the impact of copper toxicity on soil ecosystems and barley growth: Identification of robust indicators. *Environ. Monit. Assess.* **2025**, *197*, 563.
16. Azhar, U.; Ahmad, H.; Shafqat, H.; Babar, M.; Munir, H.M.S.; Sagir, M.; Arif, M.; Hassan, A.; Rachmadona, N.; Rajendran, S.; et al. Remediation techniques for elimination of heavy metal pollutants from soil: A review. *Environ. Res.* **2022**, *214*, 113918.
17. Jeyakumar, P.; Debnath, C.; Vijayaraghavan, R.; Muthuraj, M. Trends in bioremediation of heavy metal contaminations. *Environ. Eng. Res.* **2023**, *28*, 220631.
18. Mohapatra, D.; Rath, S.K.; Mohapatra, P.K. Soil fungi for bioremediation of pesticide toxicants: A perspective. *Geomicrobiol. J.* **2022**, *39*, 352–372.
19. Dhalaria, R.; Kumar, D.; Kumar, H.; Nepovimova, E.; Kuča, K.; Torequl Islam, M.; Verma, R. Arbuscular mycorrhizal fungi as potential agents in ameliorating heavy metal stress in plants. *Agronomy* **2020**, *10*, 815.
20. Bi, Y.; Xiao, L.; Liu, R. Response of arbuscular mycorrhizal fungi and phosphorus solubilizing bacteria to remediation abandoned solid waste of coal mine. *Int. J. Coal Sci. Technol.* **2019**, *6*, 603–610.
21. Hassan, N.; Khalaphallah, R. Root rot/wilt incidence of *Phaseolus vulgaris* and antagonistic effect of *Trichoderma* spp. and *Aspergillus terreus* isolated from rhizosphere soil. *SVU Int. J. Agric. Sci.* **2023**, *5*, 26–37.
22. Wijeratne, E.K.; Turbyville, T.J.; Zhang, Z.; Bigelow, D.; Pierson, L.S.; VanEtten, H.D.; Whitesell, L.; Canfield, L.M.; Gunatilaka, A.L. Cytotoxic constituents of *Aspergillus terreus* from the rhizosphere of *Opuntia versicolor* of the Sonoran desert. *J. Nat. Prod.* **2003**, *66*, 1567–1573.
23. Gao, H.; Guo, W.; Wang, Q.; Zhang, L.; Zhu, M.; Zhu, T.; Gu, Q.; Wang, W.; Li, D. Aspulvinones from a mangrove rhizosphere soil-derived fungus *Aspergillus terreus* Gwq-48 with anti-influenza A viral (H1N1) activity. *Bioorg. Med. Chem. Lett.* **2013**, *23*, 1776–1778.
24. Akbar, M.; El-Sabrout, A.M.; Shokralla, S.; Mahmoud, E.A.; Elansary, H.O.; Akbar, F.; Din, B.U.; Haroon, U.; Ali, M.; Saleem, H.; et al. Preservation and recovery of metal-tolerant fungi from industrial soil and their application to improve germination and growth of wheat. *Sustainability* **2022**, *14*, 5531.
25. Afzal, S.; Chaudhary, N.; Singh, N.K. Role of soluble sugars in metabolism and sensing under abiotic stress. In *Plant Growth Regulators: Signalling Under Stress Conditions*; Springer: Cham, Switzerland, 2021; pp. 305–334.
26. Cobbett, C.; Goldsbrough, P. Phytochelatins and metallothioneins: Roles in heavy metal detoxification and homeostasis. *Annu. Rev. Plant Biol.* **2002**, *53*, 159–182.
27. Rahim, W.; Khan, M.; Al Azzawi, T.N.I.; Pande, A.; Methela, N.J.; Ali, S.; Imran, M.; Lee, D.S.; Lee, G.M.; Mun, B.G.; et al. Exogenously applied sodium nitroprusside mitigates lead toxicity in rice by regulating antioxidants and metal stress-related transcripts. *Int. J. Mol. Sci.* **2022**, *23*, 9729.
28. Demidchik, V.; Straltsova, D.; Medvedev, S.S.; Pozhvanov, G.A.; Sokolik, A.; Yurin, V. Stress-induced electrolyte leakage: The role of K^+^-permeable channels and involvement in programmed cell death and metabolic adjustment. *J. Exp. Bot.* **2014**, *65*, 1259–1270.
29. Li, H.; Guo, C.; Su, F.; Li, L. Effects of cadmium on superoxide dismutase activity in reed leaves. *Nat. Environ. Pollut. Technol.* **2022**, *21*, 1389–1393.
30. Kong, W.; Liu, F.; Zhang, C.; Zhang, J.; Feng, H. Non-destructive determination of malondialdehyde (MDA) distribution in oilseed rape leaves by laboratory scale NIR hyperspectral imaging. *Sci. Rep.* **2016**, *6*, 35393.
31. Chi, M.H.; Park, S.Y.; Lee, Y.H. A quick and safe method for fungal DNA extraction. *Plant Pathol. J.* **2009**, *25*, 108–111.
32. Asim, S.; Hussain, A.; Murad, W.; Hamayun, M.; Iqbal, A.; Rehman, H.; Tawab, A.; Irshad, M.; Alataway, A.; Dewidar, A.Z.; et al. Endophytic *Fusarium oxysporum* GW controlling weed and an effective biostimulant for wheat growth. *Front. Plant Sci.* **2022**, *13*, 922343.
33. Tamura, K.; Dudley, J.; Nei, M.; Kumar, S. MEGA4: Molecular evolutionary genetics analysis (MEGA) software version 4.0. *Mol. Biol. Evol.* **2007**, *24*, 1596–1599.
34. Alici, E.H.; Arabaci, G. Determination of SOD, POD, PPO and CAT enzyme activities in *Rumex obtusifolius* L. *Annu. Res. Rev. Biol.* **2016**, *11*, 1–7.
35. Bajji, M.; Kinet, J.M.; Lutts, S. The use of the electrolyte leakage method for assessing cell membrane stability as a water stress tolerance test in durum wheat. *Plant Growth Regul.* **2002**, *36*, 61–70.
36. Velikova, V.; Yordanov, I.; Edreva, A. Oxidative stress and some antioxidant systems in acid rain-treated bean plants: Protective role of exogenous polyamines. *Plant Sci.* **2000**, *151*, 59–66.
37. Heath, R.L.; Packer, L. Photoperoxidation in isolated chloroplasts. I. Kinetics and stoichiometry of fatty acid peroxidation. *Arch. Biochem. Biophys.* **1968**, *125*, 189–198.
38. Dubois, M.; Gilles, K.A.; Hamilton, J.K.; Rebers, P.T.; Smith, F. Colorimetric method for determination of sugars and related substances. *Anal. Chem.* **1956**, *28*, 350–356.
39. Bradford, M.M. A rapid and sensitive method for the quantitation of microgram quantities of protein utilizating the principle of protein dyes binding. *Anal. Biochem.* **1976**, *72*, 248–254.
40. Larone, D.H. *Medically Important Fungi: A Guide to Identification*; Elsevier: New York, NY, USA, 1987.

The authors confirm that the entire reference list has been carefully reviewed and corrected. All references have been verified for accuracy, relevance, and proper attribution to the corresponding statements in the manuscript. The authors state that the scientific conclusions are unaffected. This Correction was approved by the Academic Editor. The original publication has also been updated.
