# Supplementary material for: Rhizofungus Aspergillus terreus Mitigates Heavy Metal Stress-Associated Damage in Triticum aestivum L
Source: Plants (Basel). 2024 Sep 21;13(18):2643. doi: 10.3390/plants13182643 (PMC11435276; doi:10.3390/plants13182643)
Supplement: Supplementary file 1 [file plants-13-02643-s001.zip › plants-3131910-supplementary.pdf]

plants

MDPI

Supplementary Materials

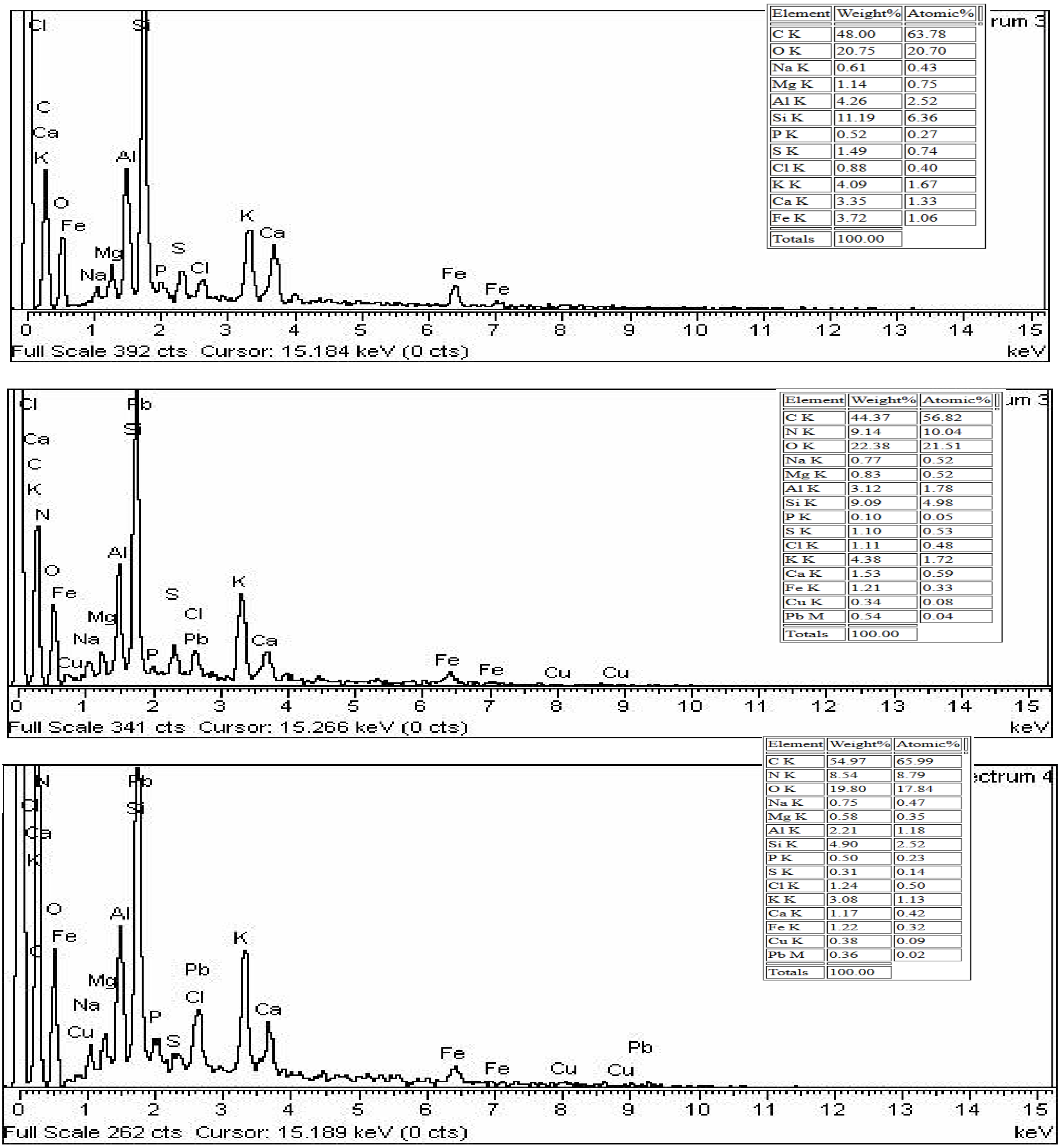

A

Element	Weight%	Atomic%
C K	48.00	63.78
O K	20.75	20.70
Na K	0.61	0.43
Mg K	1.14	0.75
Al K	4.26	2.52
Si K	11.19	6.36
P K	0.52	0.27
S K	1.49	0.74
Cl K	0.88	0.40
K K	4.09	1.67
Ca K	3.35	1.33
Fe K	3.72	1.06
Totals	100.00	

B

Element	Weight%	Atomic%
C K	44.37	56.82
N K	9.14	10.04
O K	22.38	21.51
Na K	0.77	0.52
Mg K	0.83	0.52
Al K	3.12	1.78
Si K	9.09	4.98
P K	0.10	0.05
S K	1.10	0.53
Cl K	1.11	0.48
K K	4.38	1.72
Ca K	1.53	0.59
Fe K	1.21	0.33
Cu K	0.34	0.08
Pb M	0.54	0.04
Totals	100.00	

C

Element	Weight%	Atomic%
C K	54.97	65.99
N K	8.54	8.79
O K	19.80	17.84
Na K	0.75	0.47
Mg K	0.58	0.35
Al K	2.21	1.18
Si K	4.90	2.52
P K	0.50	0.23
S K	0.31	0.14
Cl K	1.24	0.50
K K	3.08	1.13
Ca K	1.17	0.42
Fe K	1.22	0.32
Cu K	0.38	0.09
Pb M	0.36	0.02
Totals	100.00	

Figure S1. The energy dispersive x-ray (EDX) image of the root tissue shows the composition of elements, in absence and presence of rhizo-fungus colonization. (A) EDX of non-inoculated root of a *T. aestivum* plant. (B) EDX of the root section of *T. aestivum* plant under induced Pb and Cu stress. (C) EDX of root sample, inoculated with rhizo-fungus.
